# Effects of Chitosan on *Clostridium perfringens* and Application in the Preservation of Pork Sausage

**DOI:** 10.3390/md18020070

**Published:** 2020-01-22

**Authors:** Shun-Hsien Chang, Ching-Hung Chen, Guo-Jane Tsai

**Affiliations:** 1 Institute of Food Safety and Risk Management, National Taiwan Ocean University, Keelung 20224, Taiwan; lewis@mail.ntou.edu.tw; 2 Department of Food Science, National Taiwan Ocean University, Keelung 20224, Taiwan; heeves_chen@tty.com.tw; 3 Center for Marine Bioenvironment and Biotechnology, National Taiwan Ocean University, Keelung 20224, Taiwan

**Keywords:** chitosan, antibacterial activity, *Clostridium perfringens*, pork sausage

## Abstract

The effects of chitosan with 95% deacetylation degree (DD95) on the spore germination, cell proliferation, and heat resistance of *Clostridium perfringens* CCRC 10,648 and CCRC 13,019 were investigated, and its application on pork sausage with sodium nitrite reduction was also evaluated. DD95 chitosan can strongly reduce the heat resistance of both strains. The *D*_80_ and *D*_100_ values for strain CCRC 13,019 decreased from 40.98 and 4.64 min to 39.21 and 3.26 min, respectively, as a result of adding 250 ppm DD95; meanwhile, addition of chitosan decreased the *D*_80_ and *D*_100_ values for CCRC 10,648 from 41.15 and 6.46 min to 39.52 and 3.78 min, respectively. In pork sausage, addition of 3000 ppm DD95 chitosan considerably slowed down the bacterial proliferation and volatile basic nitrogen production. There were no significant differences in color (L* and b* values), shearing force, and hardness in the pork sausages with or without DD95 chitosan during storage at 4 and 25 °C. However, the addition of DD95 chitosan in pork sausage significantly retarded the decrease of the a* value. Therefore, DD95 chitosan could reduce the concentration of sodium nitrite required in pork sausages for color retention.

## 1. Introduction

The meat processing industry regularly faces many serious challenges regarding the safety and hygiene of various products [1,2,3]. The pathways for pathogens’ transmission into the product could be from throughout handling processes or from the carcass surface [4,5]; however, decontamination or sterilization of the carcass body is very difficult to perform using currently applied antimicrobial agents. Moreover, most commonly used preservatives and antimicrobial agents have a chemical and synthetic nature, giving them many potential side effects and risks on the health of consumers [6,7,8]. The meat industry continues to seek natural preservatives as antimicrobial agents.

Chitosan, a partially deacetylated chitin (poly-β-(1→4)*N*-acetyl-d-glucosamine) is found in shrimp shells and fungi [9]. This polysaccharide has attracted attention as a biomedical material because of its biocompatibility and various biological activities, including immune-enhancing [10], anti-inflammatory [11], antibacterial [6], and antitumor activities [12]. Because of its excellent antibacterial activity, chitosan has been widely used for food protection [13,14,15,16]. Several studies have shown that chitosan’s molecular weight (MW) is a crucial factor in its antimicrobial properties, although equivocal results in terms of the correlation between the antibacterial properties and MWs of chitosan have also been observed [17,18]. Our previous report [6] concluded that the correlation between chitosan MW and its antibacterial properties was dependent on the pH value of the reaction mixture.

*Clostridium perfringens*, an anaerobic spore-forming foodborne pathogen, is widely distributed in various foods, such as meat, seafood, and vegetable products. Due to the heat resistance of *C. perfringens* spores, spores may survive the heating treatment, and then germinate and proliferate in food. After the contaminated food is ingested, this pathogen secretes an enterotoxin in the intestine, which changes the permeability of the cell membrane and causes diarrheal syndrome [19] or even death [20]. Therefore, methods for controlling *C. perfringens*, especially its spores, in various meat products are necessary [21]. Addition of nitrite derivatives, including sodium and potassium nitrite, is the most effective method for controlling the growth of vegetative cells and spores of *Clostridium* species including *C. perfringens* in processed meats. In addition, nitrite also brings a characteristic red color in processed meats [22].

However, the International Agency for Research on Cancer (IARC) reported that consumption of processed meats including sausages may increase the risk for colorectal cancer, and accordingly classified processed meats as carcinogenic to humans. Nitrates and nitrites used as additives in processed meats are sources of *N*-nitroso compounds (NOCs), which have long been known as carcinogenic [23]. Several studies have attempted to develop new methods to inactivate the spores of *Clostridium* as a way to decrease or even avoid the addition of nitrites in processed meats [24].

In this study, we prepared shrimp chitosan with 95% deacetylation (DD95) and demonstrated that DD95 has a strong antibacterial activity against *C. perfringens* and could reduce the heat resistance of *C. perfringens* spores. Moreover, DD95 in pork sausage stored at 4 and 25 °C effectively retarded the increase of both total bacterial and *C. perfringens* counts (CPC), as well as volatile basic nitrogen values. In addition, the synergistic effect of DD95 and sodium nitrite for a favored color development in pork sausage was observed. Thus, the addition of DD95 in pork sausage was able to reduce the amount of sodium nitrite required and extended the shelf life of pork sausage.

## 2. Result and Discussion

### 2.1. Antibacterial Activity of Chitosan Against C. perfringens

**Figure 1:**
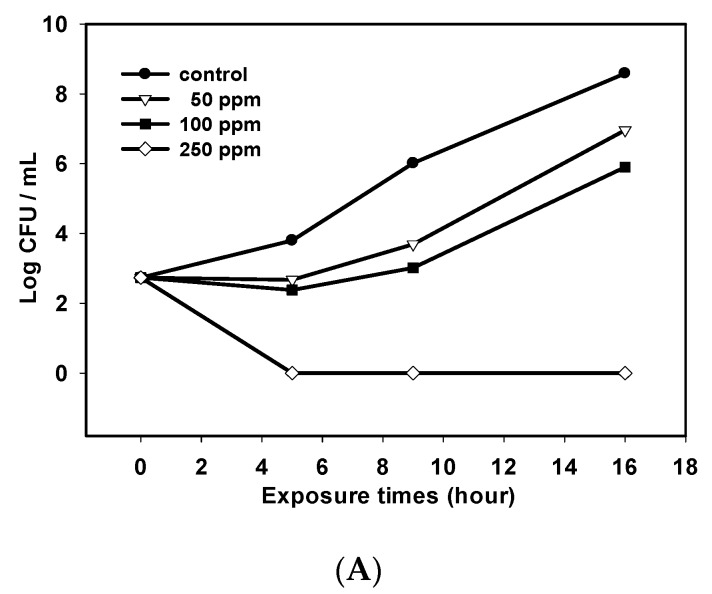

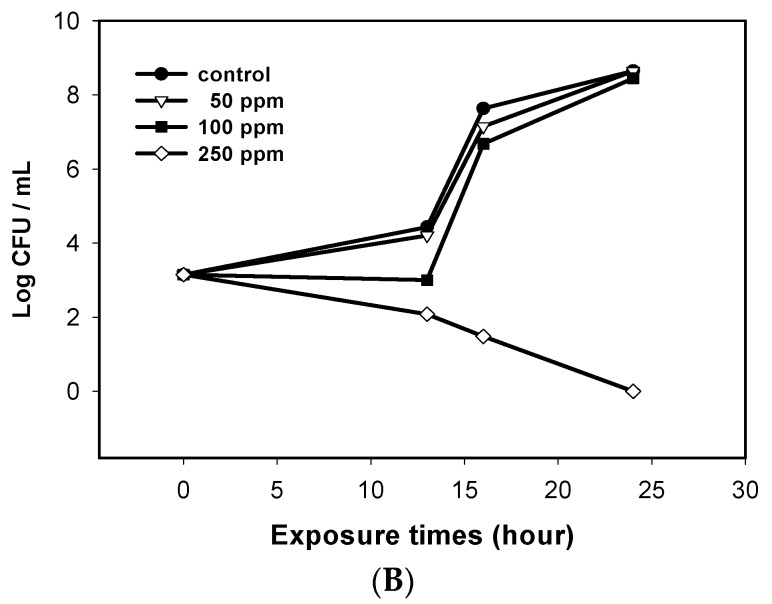
Survival for *Clostridium perfringens* CCRC 13,019 (**A**) and CCRC 10,648 (**B**) in liver brain broth (LIB) containing various concentrations of chitosan after incubation at 37 °C anaerobically.

In this study, chitosan with a molecular weight of 220 kDa and 95% degree of deacetylation was prepared from shrimp chitin [6]. The antibacterial effect of DD95 against the vegetative cells of *C. perfringens* CCRC 13,019 and CCRC 10,648 in liver infusion broth (LIB) at 37 °C anaerobically is shown in Figure 1. DD95 at the dosages of 50 and 100 ppm only retarded the growth of *C. perfringens*. However, DD95 at 250 ppm had a bactericidal effect against this pathogen. The strain CCRC 13,019 (Figure 1A) was more susceptible to DD95 compared with the strain CCRC 10,648 (Figure 1B), as evidenced by the exposure time of 5 h for the former and 24 h for the latter, with no survival being observed at 250 ppm DD95.

### 2.2. Effect of Chitosan on Decimal Reduction Time of C. perfringens Spores

The outbreak of *C. perfringens* poisoning is generally initiated by *C. perfringens* spores that survive the cooking process and are germinated in food, eventually producing enterotoxins in the intestinal tract after ingestion [16]. Therefore, reduction of heat resistance of the spores to decrease spore survival during heating is a preventative approach for *C. perfringens* poisoning [16]. The effect of DD95 at 250 ppm on the decimal reduction time (*D* value) of tested *C. perfringens* strains during heating are shown in Table 1. The *D* values at 80 °C (*D*_80_) for spores of *C. perfringens* CCRC 10,648 and CCRC 13,019 were 41.15 and 40.98 min, respectively. In the presence of 250 ppm DD95, *D*_80_ values decreased to 39.52 and 39.21 min, respectively. Meanwhile, the *D*_100_ values of these spores decreased from 6.46 and 4.64 min to 3.78 and 3.26 min, respectively, if 250 ppm DD95 was added.

### 2.3. Applications of Chitosan in Pork Sausage

**Figure 2:**
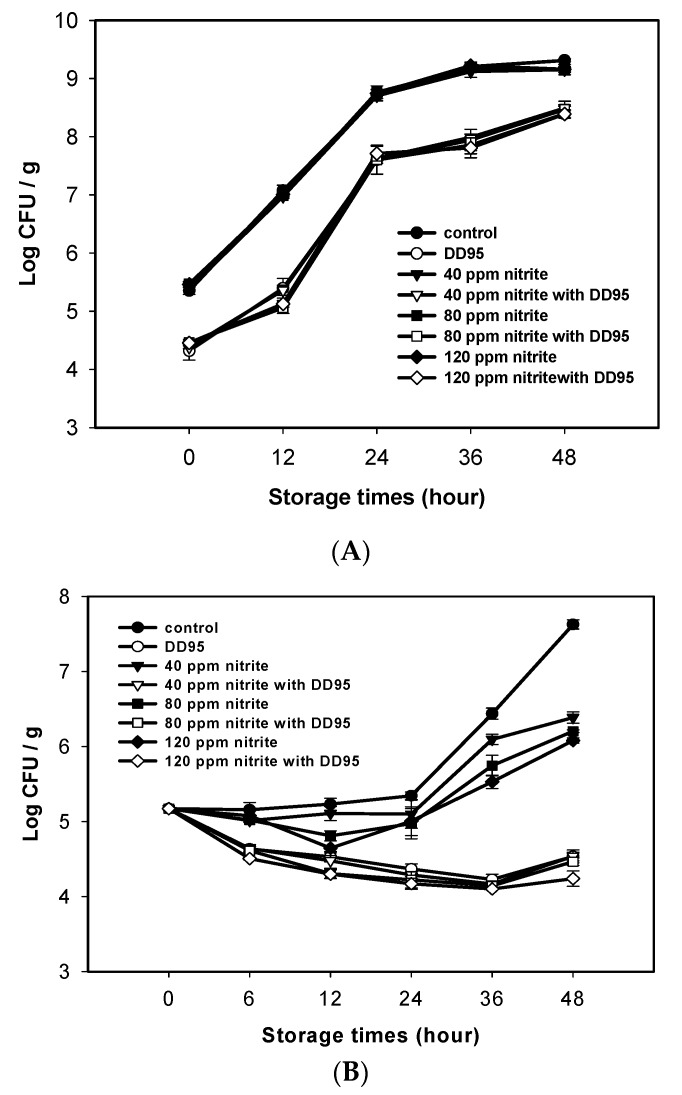
Changes in total aerobic counts (**A**) and *C. perfringens* counts (**B**) of pork sausage containing nitrite and (or) 3000 ppm DD95 chitosan during storage at 25 °C. ● and ○, 0 ppm sodium nitrite; ▼ and ▽, 40 ppm sodium nitrite; ■ and □, 80 ppm sodium nitrite; ◆ and ◇, 120 ppm sodium nitrite. Empty symbols, with 3000 ppm DD95; solid symbols, without DD95.

**Figure 3:**
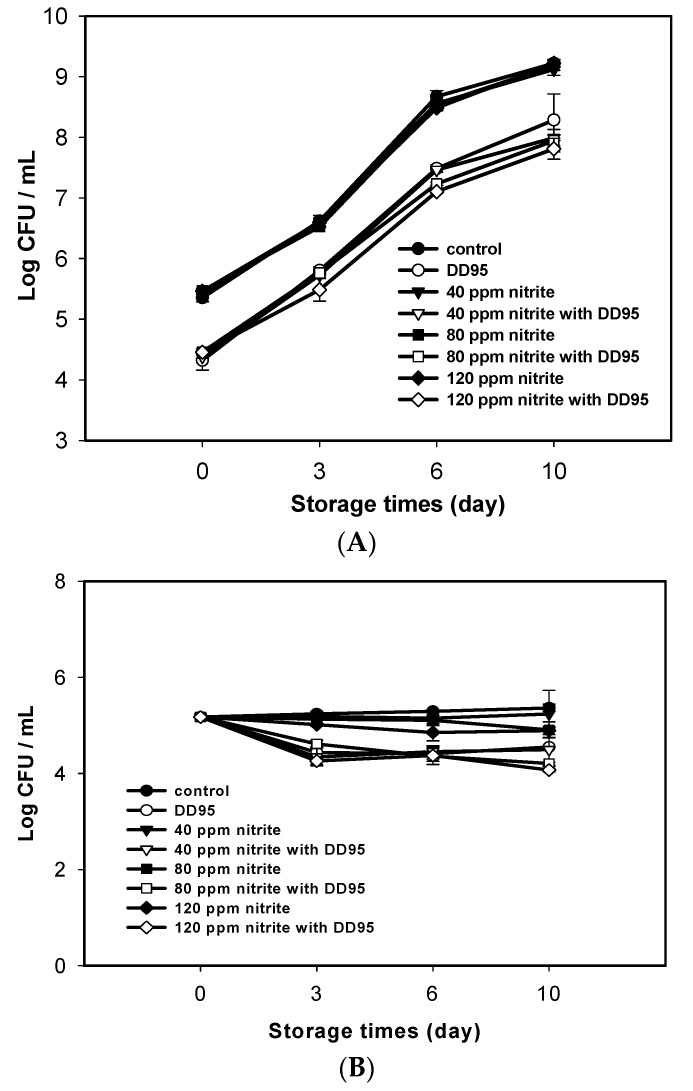
Changes in total aerobic counts (**A**) and *C. perfringens* counts (**B**) of pork sausage containing nitrite and (or) 3000 ppm DD95 chitosan during storage at 4 °C. ● and ○, 0 ppm sodium nitrite; ▼ and ▽, 40 ppm sodium nitrite; ■ and □, 80 ppm sodium nitrite; ◆ and ◇, 120 ppm sodium nitrite. Empty symbols, with 3000 ppm DD95; solid symbols, without DD95.

Ground pork containing various amounts of sodium nitrite was spiked with *C. perfringens* CCRC 13,019 spores to have the initial density of approximately 10^4^ spore/g for the preparation of pork sausages. For the experimental group, DD95 was added to have the final concentration of 3000 ppm in sausage. The sausages with or without DD95 were stored at 25 and 4 °C. Changes in the total aerobic count (TAC) and *C. perfringens* count (CPC) of pork sausage samples during storage at 25 and 4 °C are shown in Figure 2 and Figure 3. The changes in TAC of sausage samples stored at 25 °C for 48 h were quite similar among the control sample (without sodium nitrite and DD95) and samples containing sodium nitrite only (40–120 ppm); meanwhile, the addition of 3000 ppm DD95 substantially decreased the increase of TAC, regardless of whether or not sodium nitrite was added (Figure 2A). Sodium nitrite alone in the sausage stored at 25 °C was able to retard the increase of CPC, and a dose-dependent effect was observed. The CPC for sausages containing DD95 gradually decreased during storage, regardless of whether or not sodium nitrite was added (Figure 2B).

Similar results were observed for sausage samples stored at 4 °C for 10 days (Figure 3). Sodium nitrite alone did not retard the increase of TAC in the sausage samples, whereas DD95 could effectively decrease the increase of TAC (Figure 3A). The dose-dependent effect of sodium nitrite alone on decreasing CPC was also observed, and this CPC decreasing effect was further enhanced by DD95 and sodium nitrite (Figure 3B).

According to Kanner et al. [25], the mechanism of nitrite inhibition in *C. perfringens* is attributable to the destruction of iron–sulfur enzymes such as ferredoxin by nitric oxide (NO), transferred from nitrite by the ferrous ion in meat, and thus inhibition of the synthesis of adenosine triphosphate (ATP). A similar nitrite inhibition effect on other *Clostridium* species, such as *Clostridium botulinum* and *Clostridium sporogenes*, was also reported [26,27]. However, the nitrite effect on *C. perfringens* was almost diminished in the presence of DD95 in sausage (Figure 2B and Figure 3B), probably because of the much stronger activity of DD95 against *C. perfringens* at 3000 ppm, which overwhelmed the inhibition effect of nitrite at 40–120 ppm. Moreover, the iron absorption activity of chitosan may prevent NO production from nitrite [28]. This merits further investigation in the future. The aerobes seemed more resistant to NO produced from nitrite at the dosages tested, probably because of more antioxidant enzymes or compounds present in aerobes to attenuate the NO effect. Therefore, it was observed that nitrite had only a slight effect on TAC in sausages during storage in this study.

### 2.4. Color and Color Difference Measurement

Changes in color (L*, a*, and b* values) of pork sausage samples during storage at 25 °C for 48 h and 4 °C for 10 days are shown in Table 2 and Table 3, respectively. In general, both L* and b* values did not significantly change for all tested samples during storage at 25 °C, and there were no significant differences in L* values among all tested sausages, regardless of whether or not sodium nitrite and/or DD95 were/was added (Table 2).

The a* value (representing the red color) for the control sausage (without nitrite and DD95) was substantially decreased with the increase in incubation time at 25 °C, from 7.18 ± 0.45 at the beginning to 2.76 ± 0.53 at the end of storage. At the beginning, the addition of sodium nitrite at 40–120 ppm did not significantly change the a* value in sausage without DD95. The retention of a* value was significantly enhanced with the increasing amount of nitrite added during storage. After 48 h of incubation, the a* values for sausages containing 80 and 120 ppm nitrite were significantly higher than those for sausages containing 0 and 40 ppm nitrite. The a* value was further enhanced by DD95 addition in nitrite-containing sausage. The sausage containing DD95 and 120 ppm nitrite had the highest a* value after incubation for 48 h. In addition, at this moment there was no significant difference in a* value between the sausage containing 120 ppm nitrite only and the sausage containing DD95 and 40 ppm nitrite (Table 2).

Similarly, all L* values were not significantly different for all sausage samples during storage at 4 °C (Table 3). Although there was some variation in the b* value of sausage samples during storage, these b* values were still quite similar (in the range of 8.97–10.45) after incubation for 10 days. For the control sausage (without DD95 and nitrite), the a* value significantly decreased with the increase in incubation time. Nitrite addition, especially at 80 and 120 ppm, substantially increased the a* value after incubation for 10 days. There was no significant difference in a* value between the sausage containing 120 ppm nitrite only and the sausage containing DD95 and 40 ppm nitrite (Table 3).

**Figure 4:**
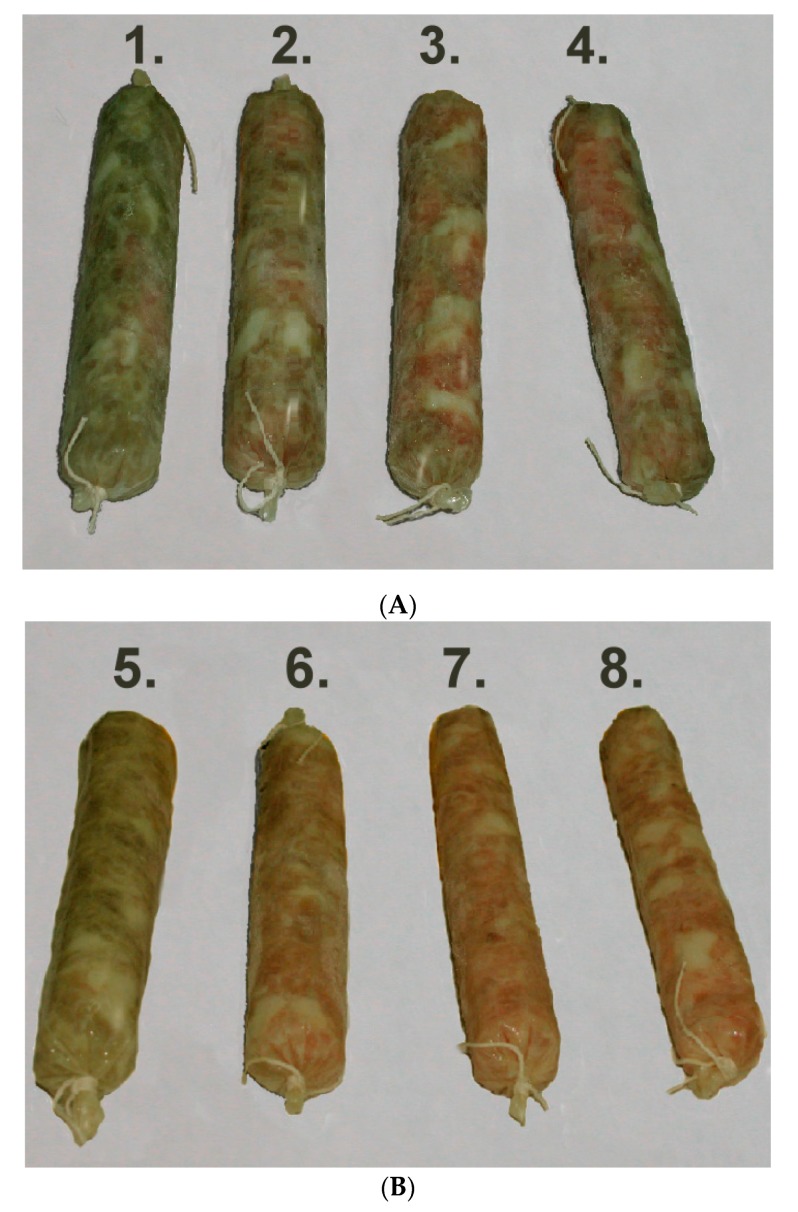
Appearance of pork sausage containing various concentrations of sodium nitrite only (**A**) and sodium nitrite plus 3000 ppm chitosan (**B**) after storage at 25 °C for 2 days. No. 1, no. 2, no. 3, and no. 4, containing 0, 40, 80, and 120 ppm nitrite only, respectively; no. 5, no. 6, no. 7, and no. 8, containing chitosan plus 0, 40, 80, and 120 ppm nitrite, respectively.

After storage at 25 °C for 48 h, the appearances of pork sausages containing 0–120 ppm sodium nitrite and with/without 3000 ppm DD95 are shown in Figure 4. The control sausage (without nitrite and DD95) became greenish. The attenuation of green color and increase of red color for sausages with increasing concentration of sodium nitrite were observed (Figure 4A). Moreover, less green color for sausage with DD95 only (sample no. 5 in Figure 4) was observed in comparison with the control sausage (sample no. 1). The light red color for sausages with DD95 and nitrite (40–120 ppm) was observed (Figure 4B). In addition, a similar or even better appearance for the sausages containing DD95 and 40 ppm nitrite (sample no. 6) was observed in comparison with that for the sausage containing 120 ppm nitrite only (sample no. 4). The color appearance of sausages shown here correlated well with the a* values shown in Table 2.

In this study, except for sodium nitrite and/or DD95, we did not add any other curing agents such as salt and sugar to eliminate the probable combination effects of these curing agents with nitrite and DD95 on color development in pork sausage. It is well known that the oxidation of deoxymyoglobin (red color) to metmyoglobin (brown color) in meat causes the decrease of a* value. The nitrosomyoglobin formation after nitrite reaction with myoglobin results in red color formation [29]—that is, it increased the a* value. The antioxidant activity of chitosan may retard the oxidation of myoglobin [30]. Accordingly, the higher a* value was obtained in sausages containing DD95 after storage at 25 and 4 °C in this study.

In addition, sulfmyoglobin formation by H_2_S induced by some putrefactive bacteria including *Pseudomonas* spp. [31] causes the greenish color of meat. This study clearly demonstrates that the addition of DD95 in sausage during storage not only retards the increase of TAC, because of its strong antibacterial activity [18], but also effectively prevents green color formation in sausages.

Owing to the carcinogenic potential of nitrite in sausages, the consumers’ interest in “synthetic nitrite-free” meat products is continuously increasing nowadays [31]. As a result, several studies have tried to use natural ingredients, such as potato paste and cured brine, to replace nitrite. However, lower L* and a* values were obtained for these products, compared to sausage added with sodium nitrite [32,33].

### 2.5. Volatile Basic Nitrogen (VBN)

**Figure 5:**
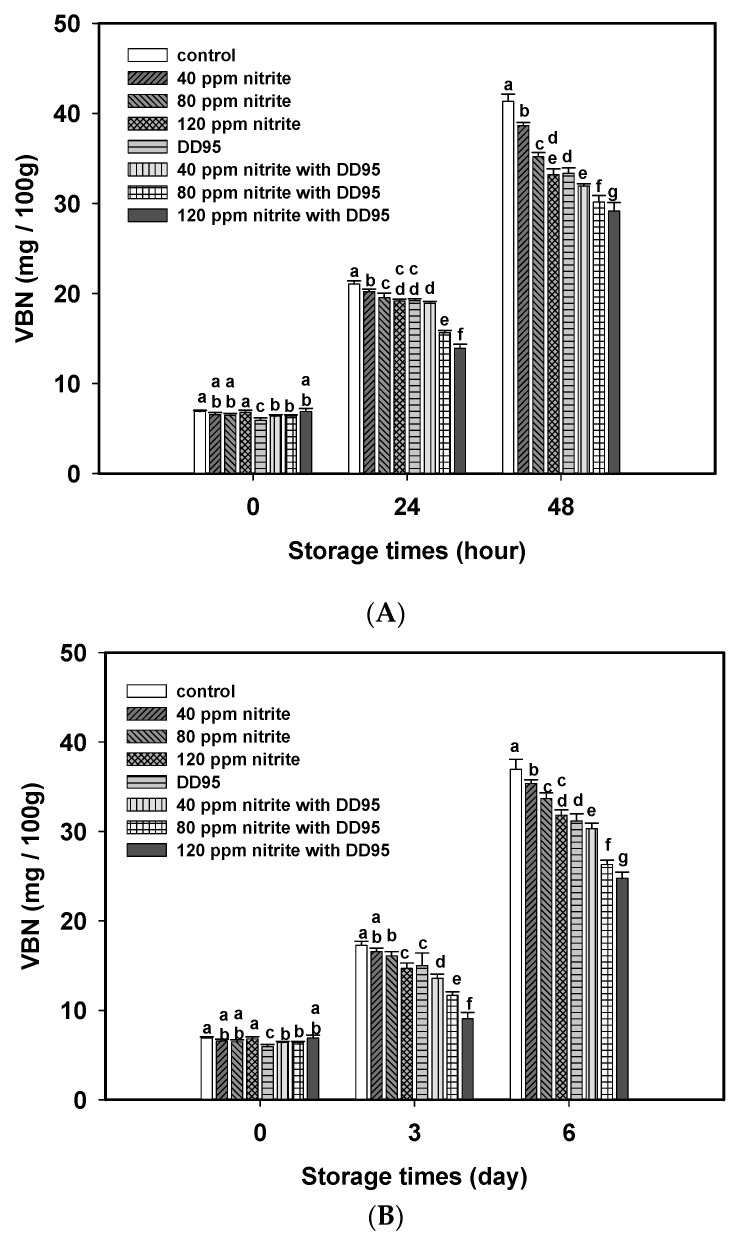
Changes in the volatile basic nitrogen (VBN) of pork sausage containing various concentrations of sodium nitrite and/or 3000 ppm chitosan (DD95) during storage at 25 °C (**A**) and 4 °C (**B**).

In the meat industry, the VBN value is used to indicate the level of putrefaction. As shown in Figure 5, the VBN values in sausages usually increased during storage at 25 °C (Figure 5A) and 4 °C (Figure 5B). Nitrite addition at 40–120 ppm significantly retarded the increase of VBN values, and a dose-dependent response was observed. The VBN values were further decreased by adding both DD95 and nitrite in sausage. We also observed that VBN values between the sausage with DD95 and 40 ppm nitrite and the sausages with 120 ppm nitrite only were quite similar after storage at 25 °C for 48 h (Figure 5A) and 4 °C for 6 days (Figure 5B). In addition, the shearing force and hardness of pork sausages with or without DD95 and nitrite during storage at 25 and 4 °C were not significantly different (data not shown).

## 3. Material and Methods

### 3.1. Bacterial Strains and Chemicals

*C. perfringens* CCRC 10,648 and CCRC 13,019 were purchased from the Biosources Collection and Research Center (Hsinchu, Taiwan, China). Acetic acid, acetonitrile, dimethyl sulfoxide (DMSO), glycerol, methanol, and sodium hydroxide (NaOH) were purchased from Fluka (Garage Gmbh, Buchs, Switzerland). Meanwhile, sodium azide (NaN_3_), phenol red, and sodium bicarbonate (NaHCO_3_) were purchased from Sigma Chemical Co. (Gillingham, UK). Cellulase (3000 U/g) from *Trichoderma viride* was purchased from Challenge Bioproducts Co., Ltd. (Taichung, Taiwan, China), whereas chitin powder was obtained from Applied Chemical Co., Ltd. (Kaohsiung, Taiwan, China). LIB, plate count agar (PCA), tryptose–sulfite–cycloserine (TSC) agar base, bacto agar, proteose peptone no. 3, yeast extract, and 50% egg yolk saline were supplied by Becton Dickinson (Sparks, MD, USA).

### 3.2. DD95 Chitosan Preparation

On the basis of the method described by Chang, Lin, Wu and Tsai [6], chitosan with 95.0% deacetylation degree, as measured using the colloid titration method [34], was obtained after deacetylation of a shrimp chitin powder suspension in 50% NaOH (1.0 g chitin per 13 mL NaOH) at 140 °C for 1 h. DD95 chitosan MW was 300 kDa, as determined by size-exclusion high-performance liquid chromatography using a column packed with TSKgel G4000 PWXL and G5000 PWXL [35].

### 3.3. Culture Conditions

*C. perfringens* CCRC 10,648 and CCRC 13,019 were stored in LIB containing 50% sterile glycerol at −80 °C. To prepare the bacteria cultures, the strains stored at −80 °C were inoculated into 50 mL LIB and anaerobically incubated at 37 °C for 18–24 h. All strains were subcultured twice in LIB at 37 °C for 18–24 h, and the cultures were diluted to 1.0 × 10^8^ CFU/mL with sterile 0.1% peptone water before use.

### 3.4. Spore Preparation

Briefly, 1 mL of the 18–24 h *C. perfringens* culture in LIB was added in 9.0 mL of the modified Ducan–Strong (DS) medium [36], and incubated for 18–24 h under anaerobic conditions. Next, 1 mL of the cultivated DS medium broth was added into 9 mL fresh modified DS medium and heated at 70 °C for 20 min, then incubated anaerobically for 20–24 h; the process was repeated twice. Cultivated broths were subsequently placed under centrifugation at 1500× *g* for 30 min; precipitates were washed under suspension in 10 mL phosphate-buffered saline (PBS) and heated at 70 °C for 20 min to inactivate the cells, and then spores were collected and stored at 4 °C until use.

### 3.5. Antibacterial Test

A 1% chitosan stock solution was prepared by adding 0.2 g of DD95 chitosan to 10 mL distilled water, sterilizing at 121 °C for 15 min, and then adding 10 mL sterile 0.2 N HCl. In 50 mL flasks containing 10 mL of LIB, DD95 chitosan in 0.1 N HCl was added to obtain concentrations of 50, 100, and 250 ppm. Next, 1 mL of *C. perfringens* spore suspension was added into a flask to an initial spore density of ca. 10^3^ CFU/mL, and incubated at 37 °C for 24 h under anaerobic conditions. Then, 0.1 mL aliquots of decimal dilutions of LIB culture were spread onto LIB agar plates, which were incubated under anaerobic conditions at 37 °C for 24 h before colonies were counted. For evaluation of chitosan against spores, spores of two strains were collected into PBS added with 250 ppm DD95 chitosan, and further incubation was performed as described in the above test. The experiments were run in triplicate.

### 3.6. Measurement of Heat Resistance of C. perfringens Spores

On the basis of the method of Tsai, Tsai, Lee and Zhong [16], 1 mL of *C. perfringens* spore suspension (1 × 10^7^ spore/mL) in sterile saline alone or in sterile saline containing 250 ppm chitosan was injected into glass microhematocrit tubes (inside diameter, 5 mm; length, 20 cm; wall thickness, 0.5 mm) and submerged in an oil bath at 90 or 100 °C. Three tubes for each strain were removed at intervals and cooled immediately in ice water. The initial spore concentration and the number of spores that survived heating were determined by spreading 0.1 mL of decimal dilutions of spore suspension on LIB agar. Colonies were counted after incubation at 37 °C for 2 days. The log transformed number of surviving spores was plotted against the corresponding heating time. Data points were fitted using the least squares method, and the reciprocal of the slope was taken as the *D* value.

### 3.7. Application of DD95 Chitosan to Pork Sausage

Raw pork sausage was added to an equal amount (w/w) of chitosan at the final concentration of 3000 and 6000 ppm. The two sausages with or without DD95 chitosan were added with *C. perfringens* strain spore cells to a density of ca. 10^4^ spore/g, then vacuum-packed and stored at 4 and 25 °C during storage until use. Then, 25 g of vacuum-packed pork sausage was poured into a sterile stomacher bag with 225 mL of 0.1% peptone water and homogenized with a stomacher (Stomacher 400 Lab Blender; Seward Medical, London, UK), and the resulting solution was diluted serially with 0.1% peptone water. The total aerobic bacterial count and the CPC for each sample were determined by spread plating 0.1 mL of sample decimal dilutions onto PCA at 37 °C for 48 h and TSC agar (250 mL TSC agar base +20 mL 50% egg yolk saline +20 mL cycloserine (0.5%)) under anaerobic conditions at 37 °C for 24 h.

### 3.8. Application of DD95 Chitosan and Nitrite for Pork Sausage

Pork sausage was added to an equal amount (w/w) of chitosan and finally to 3000 ppm of DD95 chitosan and 0, 40, 80, and 120 ppm of nitrite substitution for pork sausage. The two sausages with or without DD95 chitosan were added with *C. perfringens* strain spore cells to a density of ca. 10^4^ spore/g, then vacuum-packed and stored at 4 and 25 °C during storage until use. The survival counts were measured as described in Section 3.7.

### 3.9. Color and Color Difference Measurement

On the basis of the method described by [37,38], samples were cut into 2 cm-thick pieces and measured on the plate of the Color Difference Meter (Spectrophotometer CM-3500d; Minolta Co., Ltd., Osaka, Japan). The instrument was calibrated to standard black and white tiles before analysis. Eight pieces per treatment were evaluated, and mean values were used for replication. A medium-size aperture was used, and the measurement was duplicated. The Hunter color L*, a*, and b* values were reported through the computerized system using Spectra Magic software (version 2.11; Minolta Cyberchrom Inc. Osaka, Japan).

### 3.10. Volatile Basic Nitrogen

According to the method described by Saito et al. [39], 5 g of the sample was homogenized with 45 mL 7% trichloroacetic acid (TCA) to obtain a final concentration of 50 mL. A volume of 0.02 N boric acid as a VBN absorber was placed in the inner section of a Conway microdiffusion cell [40]. Then, 1 mL of the homogenized solution was pipetted to the Conway dish containing 1 mL saturated K_2_CO_3_ solution and allowed for reaction at 37 °C for 90 min. The boric acid solution containing indicator (methyl red and bromocresol green) was used to absorb volatile nitrogen. The solution was titrated with 0.01 N HCl. The VBN value, expressed as mg %, was calculated as follows: VBN value (mg %) = 0.02 × 14 × (*a* − *b*) × *F* × *V*/*W* × 100, where *a* is the titration volume of sample solution, *b* is the titration volume of water blank, *V* is the final volume with TCA of sample after extraction, *W* is the mass of the sample (g), and *F* is the unit of 0.02 HCl.

### 3.11. Statistical Analysis

Data were analyzed using the general linear model (GLM) of Statistical Analysis System’s Procedures (SAS Institute Inc., Cary, NC, USA) with a 5% level of significance. Means were separated using Duncan’s new multiple range test.

## 4. Conclusions

Sodium nitrite at 40–120 ppm significantly retarded the proliferation of *C. perfringens* and VBN values, and increased red color retention in pork sausage during storage. However, sodium nitrite at the tested concentration had little inhibition effect on the growth of the aerobes. DD95 chitosan clearly inhibited the growth of *C. perfringens* and reduced the heat resistance of the spore of this pathogen. The addition of DD95 chitosan effectively slowed down the increase of TAC and VBN values, preserved the red color, and inhibited the growth of *C. perfringens*. In effect, DD95 chitosan could serve as a substitute for part of the total amount of sodium nitrite used in pork sausages as a way to reduce the sodium nitrite content in pork sausages. DD95 chitosan shows strong potential as an alternative natural preservative and can help maintain the stability of color in pork sausages.

## Figures and Tables

**Table 1:** Effect of chitosan on the *D* value (min) of *C. perfringens* spores.

Heating Treatment (°C)	DD95 Conc. (ppm)	CCRC 10,648 *	CCRC 13,019
80	0	41.15	40.98
80	250	39.52	39.21
100	0	6.46	4.64
100	250	3.78	3.26

* Data are mean of the duplicate experiments. DD95: 95% deacetylation.

**Table 2:** Changes in L*, a*, and b* values of pork sausage containing chitosan (DD95) and various concentrations of sodium nitrite during storage at 25 °C.

Assay	Group ^a^	Nitrite Concentration (ppm)	Values of Pork Sausage after Following Hours of Storage ^b,c^				
			0	12	24	36	48
L	Without DD95	0	B42.2 ± 1.8a	A47.5 ± 1.6a	AB44.5 ± 2.3bc	AB46.1 ± 3.3a	AB45.4 ± 3.3a
		40	A45.6 ± 2.1a	A48.9 ± 4.9a	A47.2 ± 2.2abc	A46.4 ± 2.2a	A46.2 ± 1.6a
		80	A45.6 ± 2.1a	A44.9 ± 4.9a	A45.2 ± 5.0bc	A46.4 ± 2.2a	A46.2 ± 3.6a
		120	B44.3 ± 1.7a	AB46.5 ± 1.5a	B43.0 ± 1.5bc	AB46.1 ± 3.5a	A48.6 ± 2.1a
	DD95	0	B46.6 ± 0.7a	AB47.4 ± 1.6a	A50.6 ± 1.5c	AB48.2 ± 3.1a	A50.6 ± 2.2a
		40	A48.6 ± 4.4a	A49.6 ± 2.4a	A45.0 ± 2.1bc	A46.2 ± 0.7a	A46.4 ± 1.6a
		80	A48.8 ± 6.3a	A49.8 ± 3.3a	A48.8 ± 2.8ab	A47.9 ± 1.3a	A49.3 ± 0.9a
		120	A48.0 ± 2.5a	A46.9 ± 1.9a	A46.7 ± 2.1abc	A44.4 ± 1.2a	A48.5 ± 4.8a
a	Without DD95	0	A7.2 ± 0.5a	AB6.60 ± 1.5ab	C3.4 ± 0.8d	BC3.7 ± 3.1d	C2.8 ± 0.5e
		40	AB7.0 ± 0.3a	A7.44 ± 1.2a	A8.1 ± 0.5ab	BC5.8 ± 1.2cd	C5.3 ± 0.5d
		80	A7.0 ± 0.5a	A7.22 ± 1.4a	A7.0 ± 1.4b	A7.2 ± 1.0abc	A6.0 ± 0.7c
		120	AB6.6 ± 1.1a	AB6.84 ± 1.3a	A8.4 ± 0.5ab	B6.5 ± 1.1bc	AB6.5 ± 0.5bc
	DD95	0	A6.5 ± 0.5a	A6.26 ± 0.0ab	AB5.4 ± 1.2c	AB5.2 ± 0.7cd	B4.4 ± 0.5d
		40	B6.1 ± 1.9a	AB6.95 ± 1.6a	A8.7 ± 0.5a	A8.6 ± 0.4ab	AB7.7 ± 1.2bc
		80	B6.4 ± 1.8a	B6.42 ± 0.4ab	A8.0 ± 0.4ab	A8.3 ± 0.5ab	A8.0 ± 1.2ab
		120	B6.7 ± 0.6a	B6.61 ± 0.3b	A8.3 ± 0.8ab	A9.3 ± 0.4a	A9.4 ± 0.8a
b	Without DD95	0	A8.3 ± 0.5b	A9.48 ± 0.7a	A8.1 ± 0.3a	A8.4 ± 1.3b	A8.5 ± 0.7b
		40	A9.4 ± 0.5ab	A9.42 ± 0.9a	A10.2 ± 0.5a	A9.4 ± 0.7ab	A9.3 ± 0.4a
		80	A9.6 ± 0.8a	A9.18 ± 0.4a	A9.4 ± 0.8a	A9.2 ± 0.4ab	A9.3 ± 0.9ab
		120	A9.3 ± 0.5ab	A9.64 ± 0.4a	A9.7 ± 0.6a	A9.7 ± 0.3ab	A10.0 ± 0.8a
	DD95	0	A9.1 ± 0.5ab	A9.21 ± 0.4a	A9.8 ± 0.3a	A9.2 ± 0.8ab	A9.5 ± 0.9ab
		40	A9.8 ± 1.0a	A9.80 ± 0.2a	A9.8 ± 0.3a	A9.8 ± 0.4a	A8.9 ± 0.6ab
		80	A9.2 ± 0.7ab	A10.2 ± 1.2a	A10.0 ± 1.1a	A9.7 ± 0.46ab	A9.9 ± 0.2a
		120	A9.3 ± 0.6ab	A8.96 ± 0.4a	A9.7 ± 0.6a	A9.1 ± 0.7ab	A9.8 ± 0.9ab

^a^ DD95, 3000 ppm chitosan with 95% deacetylation degree added. ^b^ Data are mean ± standard deviation (*n* = 3). ^c^ a–c, different letters in the column for the same test item are significantly different (*p* < 0.05); A,B, different letters in the same row with are significantly different (*p* < 0.05).

**Table 3:** Changes in L*, a*, and b* values of pork sausage containing chitosan (DD95) and various concentrations of sodium nitrite during storage at 4 °C.

Assay	Group ^a^	Nitrite Concentration (ppm)	Values of Pork Sausage after Following Days of Storage ^b,c^			
			0	3	6	10
L	Without DD95	0	B42.2 ± 1.8a	AB45.3 ± 2.3abc	A47.0 ± 1.5a	A48.0 ± 1.0a
		40	A45.6 ± 2.1a	A42.8 ± 1.1c	A45.9 ± 2.9a	A47.5 ± 4.3a
		80	A45.6 ± 2.1a	A44.5 ± 3.0bc	A48.3 ± 2.1a	A45.2 ± 1.4a
		120	B44.3 ± 1.7a	A46.3 ± 2.1abc	A47.5 ± 2.7a	A44.3 ± 1.7a
	DD95	0	A46.6 ± 0.7a	A48.6 ± 1.8ab	A45.7 ± 3.5a	A47.1 ± 2.1a
		40	A48.6 ± 4.4a	A47.2 ± 4.2ab	A49.2 ± 1.6a	A45.3 ± 3.7a
		80	A48.8 ± 6.3a	A49.6 ± 2.4a	A49.8 ± 3.2a	A48.8 ± 1.7a
		120	A47.9 ± 2.5a	A46.0 ± 2.6abc	A48.0 ± 1.8a	A45.5 ± 2.4a
a	Without DD95	0	A7.18 ± 0.5a	A6.9 ± 0.4abc	AB6.2 ± 1.9a	B4.3 ± 1.6d
		40	AB6.95 ± 0.3a	A7.3 ± 0.9a	AB6.8 ± 0.7a	B5.3 ± 2.2cd
		80	A6.95 ± 0.5a	A7.5 ± 0.4ab	A7.7 ± 0.1a	A8.0 ± 0.8ab
		120	A6.62 ± 1.1a	A6.4 ± 0.7abc	A6.0 ± 1.6a	A7.8 ± 1.4abc
	DD95	0	A6.46 ± 0.5a	A6.4 ± 0.2abc	A6.2 ± 0.4a	A5.7 ± 1.2bcd
		40	A7.10 ± 1.9a	A7.5 ± 2.3bc	A7.6 ± 1.3b	A8.7 ± 1.7a
		80	A7.37 ± 1.8a	A7.2 ± 1.5bc	A7.3 ± 0.6b	A7.9 ± 0.8bcd
		120	A7.66 ± 0.6a	A8.0 ± 1.6c	A7.0 ± 0.3b	A7.9 ± 0.1d
b	Without DD95	0	A8.25 ± 0.5a	A8.9 ± 0.6b	A9.3 ± 1.3a	A9.0 ± 0.5c
		40	B9.38 ± 0.5a	B9.1 ± 0.3ab	B9.1 ± 0.7a	A10.5 ± 0.4a
		80	A9.58 ± 0.8a	A9.5 ± 0.3ab	A10.4 ± 0.5a	A10.2 ± 0.6a
		120	A9.27 ± 0.5a	A10.0 ± 0.5a	A10.0 ± 0.9a	A9.8 ± 0.3abc
	DD95	0	A9.10 ± 0.5a	A9.6 ± 0.6ab	A8.9 ± 1.3a	A9.1 ± 0.4c
		40	A9.76 ± 1.1a	A9.4 ± 0.9ab	A9.7 ± 0.5a	A10.1 ± 0.2ab
		80	A9.17 ± 0.7a	A9.5 ± 0.7ab	A9.8 ± 1.0a	A9.3 ± 0.4bc
		120	A9.26 ± 0.6a	A8.9 ± 0.5b	A9.5 ± 0.9a	A9.3 ± 0.6bc

^a^ DD95, 3000 ppm chitosan with 95% deacetylation degree added. ^b^ Data are mean ± standard deviation (*n* = 3). ^c^ a–d, different letters in the column for the same test item are significantly different (*p* < 0.05); A,B, different letters in the same row are significantly different (*p* < 0.05).
